# Review of Multiple Myeloma Genetics including Effects on Prognosis, Response to Treatment, and Diagnostic Workup

**DOI:** 10.3390/life12060812

**Published:** 2022-05-30

**Authors:** Julia Erin Wiedmeier-Nutor, Peter Leif Bergsagel

**Affiliations:** Division of Hematology and Oncology, Mayo Clinic, Phoenix, AZ 85054, USA; bergsagel.leif@mayo.edu

**Keywords:** multiple myeloma, cytogenetics, mutation, molecular genetics, targetable therapies, minimal residual disease

## Abstract

Multiple myeloma is a disorder of the monoclonal plasma cells and is the second most common hematologic malignancy. Despite improvements in survival with newer treatment regimens, multiple myeloma remains an incurable disease and most patients experience multiple relapses. Multiple myeloma disease initiation and progression are highly dependent on complex genetic aberrations. This review will summarize the current knowledge of these genetic aberrations, how they affect prognosis and the response to treatment, and review sensitive molecular techniques for multiple myeloma workup, with the ultimate goal of detecting myeloma progression early, allowing for timely treatment initiation.

## 1. Introduction

Multiple myeloma (MM) is a disorder of the monoclonal plasma cells. It is the second most common hematologic malignancy in high-income countries and its global incidence is increasing [1]. Monoclonal gammopathy of undetermined significance (MGUS) and smoldering myeloma (SMM) are considered pre-malignant states of MM. Newer therapies have improved patient outcomes; however, MM remains an incurable disease and the majority of patients experience multiple relapses. MM disease initiation and progression are highly dependent on genetic alterations, including chromosomal translocations and other structural variants (SVs) such as deletions, duplications, and insertions. Deleterious single-nucleotide variants (SNVs) are also common and can lead to MM disease progression and treatment resistance. Next-generation sequencing (NGS) and other highly sensitive technologies have highlighted the complexity of MM genetics and allowed new perspectives on the timing, origins, and detection of MM, giving further insight into the mechanisms behind MM progression and responses to treatment. Currently, there are no targeted treatments available for multiple myeloma but perhaps, through the further discovery and characterization of these genetic aberrations, targeted treatment may be possible. This review will summarize what is currently known about genetic aberrations in MM and their pre-malignant states, how MM genetics affect prognosis and the response to treatment, and, finally, summarize the highly sensitive diagnostic workup of MM made possible by molecular technologies.

## 2. Genetic Aberrations in Multiple Myeloma

### 2.1. Primary or Clonal Genetic Events in Multiple Myeloma

Primary events in MM are considered clonal events and occur early in the pathogenesis of the disease. These primary events include immunoglobulin (Ig) translocations and hyperdiploidy (HRD), which is the gain of odd-numbered chromosomes. Chromosomal translocations are mediated by errors in VDJ recombination (a process of chromosomal breakage and rejoining in developing B-cells, starting in the pre-pro-B stage and ending in the pro-B stage) and place oncogenes under the control of strong enhancers (Ig heavy chain (IgH) loci) [2,3]. The etiology of acquisition of these primary events remains unknown, but they are present early on in the disease process, for example, in SMM patients, common MM Ig translocations may be present in one-third and HRD may be present in about half of SMM patients [4]. IgH translocations increase with each stage of disease, occurring in approximately 50% of MGUS and SMM, in 60–65% of intramedullary MM, and in 70–80% of extramedullary MM [5,6,7]. 

**Figure 1:**
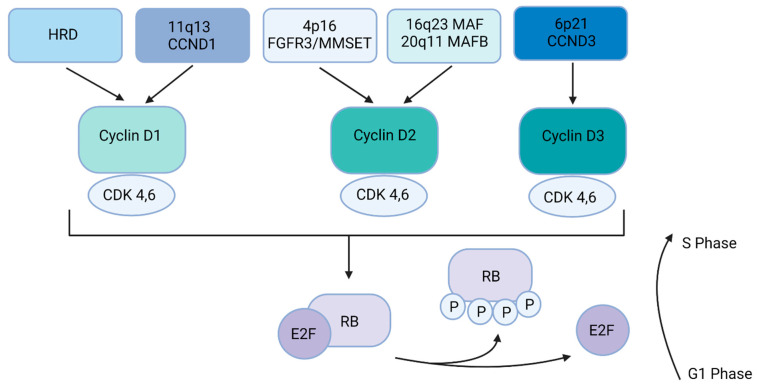
Multiple myeloma dysregulation of the RB pathway. Primary events (HRD, t(11;14), t(4;14), t(14;16), t(14; 20), and t(6;14) lead to the dysregulation of a D cyclin. Cyclin D1, cyclin D2, and cyclin D3 interact with CDK4 and CDK6 to phosphorylate RB, allowing the E2F family transcription factors to activate the G1-to-S phase transition in the cell cycle.

The five most frequent primary IgH (found on chromosome 14) translocations in MM are translocation to the long arm of chromosome 11 (11q13, t(11;14)), translocation to the short arm of chromosome 4 (4p16, (t(4;14)), translocation to the long arm of chromosome 16 (16q23, t(14;16)), translocation to the long arm of chromosome 20 (20q11, t(14;20)), and translocation to the short arm of chromosome 6 (6p21, t(6;14)) [3,4,8,9,10,11,12,13] (Table 1). In general, these translocations lead to the dysregulation of a D-group cyclin. In other patients there is inactivation of the retinoblastoma gene (RB) leading to dysregulation of G1-to-S-phase transition [3,14] (Figure 1). In fact, virtually all stages of the disease, including MGUS, SMM, and MM tumors, have dysregulated and/or increased expression of cyclin D1, cyclin D2, or cyclin D3, including translocations involving MAF, MAFB, fibroblast growth factor 3 (FGFR3), and HRD disease [3,4]. 

HRD MM is associated with the gain of odd-numbered chromosomes, including 3, 5, 7, 9, 11, 15, 19, and 21. Unlike with primary IgH translocations, the effects of HRD on MM pathogenesis have been difficult to identify [4]. The mechanism underlying HRD acquisition is also unknown, but NGS techniques have shed light on these possible mechanisms (see below). HRD MM can be classified based on the gain of chromosome 11 and CCND1 expression, where individuals with a gain of chromosome 11 have improved outcomes compared to those without a gain of chromosome 11 [15,16]. 

### 2.2. Secondary or Sub-Clonal Genetic Events in Multiple Myeloma

Secondary genetic events in MM occur against a background of primary events and are often more complex, with additional copy-number abnormalities, such as gains (duplications), losses (deletions), other translocations, and somatic mutations (deleterious single nucleotide variants), and are often associated with progression [5]. Some of the most common progression events in MM include mutations in the driver genes, such as rat sarcoma virus (*RAS)* mutations, proto-oncogene *MYC* dysregulation through SVs, and a variety of mutations that inactivate the NFKB pathway, but there are many other important genes that lead to progression [3].

The most frequent sub-clonal copy number abnormalities include deletion of the long arm of chromosome 13 (del(13q)), gain of the long arm of chromosome 1 (gain 1q), deletion of the long arm of chromosome 14 (del(14q)), deletion of the short arm of chromosome 17 (del(17p)), and deletion of the short arm of chromosome 1 (del(1p)) [1,15,17,18] (Table 2). The most common tumor suppressor genes in MM are the family with sequence similarity 46, member c (*FAM46C)*, exosome complex exonuclease (*DIS3)*, CYLD lysine 63 deubiquitinase (*CYLD)*, baculoviral IAP repeat-containing protein 2 (*BIRC2)*, baculoviral IAP repeat-containing protein 3 (*BIRC3)*, and TNF receptor-associated factor 3 (*TRAF3)*. Cyclin-dependent kinase inhibitor 2A (*CDKN2A)*, *RB1*, and tumor protein P53 (*TP53)* are other tumor suppressor genes that are important for the development and progression of MM. There is a high frequency of RAS mutations in MM, including neuroblastoma RAS viral oncogene (*NRAS)* (17–24% of cases), Kirsten rat sarcoma virus (*KRAS)* (22–27% of cases), and proto-oncogene *BRAF* (4–8% of cases, with a higher prevalence in relapsed/refractory MM (RRMM)), indicating that this pathway is critical for MM progression [15,19]. Other important mutations include those associated with the nuclear factor kappa light-chain-enhancer of activated B cells (NF-kB) activation (occurring in about 14% of MM), G1/S cell cycle transition (occurring in about 5% of MM), Ras-Raf-MEK-ERK pathway (MEK/ERK) signaling, RNA processing, and epigenetic regulators [1,15] (Table 3). Some of these driver gene alterations may also be present in SMM patients; one group found that *KRAS*, *NRAS*, *BRAF*, and the phosphatase and tensin homolog (*PTEN)* were present in about 46% of SMM patients, while *TP53* and *ATM* serine/threonine kinase were present in about 10% [2]. As the number of mutations in driver genes increases, there is an association with worse prognoses of progression-free survival (PFS) and overall survival (OS) [2,15]. 

A common and important secondary genetic event includes translocations involving *MYC*, where key driver genes and super-enhancers are juxtaposed to the *MYC* locus, leading to *MYC* dysregulation; these are often complex events [20] (Table 2). *MYC* SVs are present in approximately 40% of newly diagnosed MM (NDMM) and are common in HRD MM (~57%) [20,21]. *MYC* SVs are also present in about a quarter of NDMM with primary IgH translocations, but these can involve immunoglobulin light chain loci, Ig kappa or Ig lambda [20,21,22]. *MYC* SVs occur in about one-quarter of SMM patients but do not appear to be present in MGUS patients [21]. 

Del(17p)/*TP53* also deserves special mention, given its importance in prognostication. Del(17p) occurs in 10% of NDMM cases and is primarily monoallelic (only occurring on one allele). *TP53* mutations are rarer at presentation but can occur in 1–7% of NDMM cases [23,24]. Overall, *TP53* alterations (*TP53* mutations or del(17p)) are more prevalent in RRMM, occurring in approximately 23–45% of cases [23,25]. 

**Figure 2:**
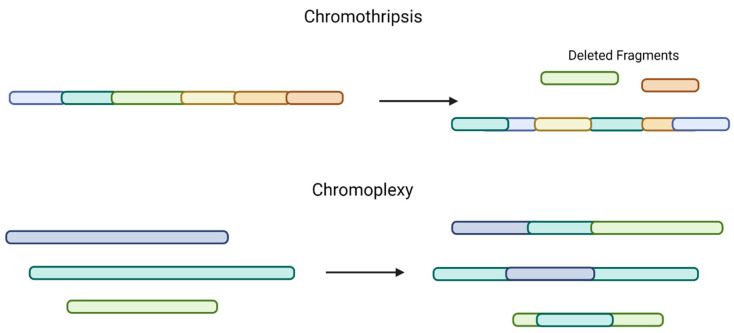
Comparison of chromothripsis and chromoplexy. Chromothripsis, a single catastrophic event, includes multiple copy-number gains, losses, and random DNA fragment joining (**top**). Chromoplexy involves structural variants across >2 chromosomes and is also associated with copy-number loss (**bottom**).

A recent study evaluated SVs in NDMM patients from the CoMMpass database (a large collection of genomic and clinical data sets of patients with NDMM) using whole-genome sequencing (WGS), whole-exome sequencing (WES), and RNA sequencing. The group found that complex SVs, including chromothripsis (a single catastrophic event that includes copy-number gains and losses), chromoplexy (interconnected structural variant breakpoints across >2 chromosomes, associated with copy-number loss), or templated insertions (copy-number gains), are common [20,26] (Figure 2). Chromothripsis was found to be present in 24% of MM patient samples, which is higher than was previously thought, and templated insertions were present in 19% of MM tumors. This higher detection rate is likely due to improved NGS techniques. The most common recurrent SVs were Ig and *MYC*. 

Co-occurrences, or oncogenic dependencies between genomic markers in MM, are common, including t(11;14) and mutations in *CCND1* (which is also associated with recurrent interferon regulatory factor 4 (*IRF4)* K123R mutations), the gain of 1q and t(4;14), t(4;14) and *TRAF3* deletion, and *FAM46C* and *CDKN2C* deletions, among others [20,27,28]. *CYLD* deletions are associated with HRD and t(11;14) but are mutually exclusive with t(4;14). Ras mutations (*NRAS*, *KRAS*, *BRAF*) can co-occur. Chromothripsis can also co-occur with the biallelic inactivation of *TP53* [20].

### 2.3. Evolution and Spatial Heterogeneity of MM, Based on Genetics

The evolutionary model of MM has been described as a Darwinian process with selective pressure from the bone marrow microenvironment [1,4,29]. NGS techniques have aided in evaluating the temporal evolution of MM. A study examined the WGS of tumor samples, collected at different time points from 30 MM patients, and used the concept of molecular time (through the integration of SVs, copy-number alterations (CNAs), and point mutations) to chronologically construct the driver events in MM, specifically, the timing of aneuploidies [20]. Although HRD is considered an early, primary event in MM, there are likely to be significant structural changes that occur between diagnosis and relapse (subclonal evolution). The group showed that in any individual patient, there were significant changes in their underlying karyotype over time, including the losses and gains of chromosomes. There was one extreme example of a patient who demonstrated an entire whole-genome duplication at relapse. In the majority of HRD patients, gains of odd chromosomes and 1q occurred as the first aneuploidies. Chromothripsis and templated insertions also appeared early as clonal events, whereas chromoplexy and focal deletions appeared later in the disease course. 

Spatial genomic heterogeneity has been described in solid tumors but has been difficult to characterize in hematologic malignancies. MM proliferates in the bone marrow, but a small number of clonal cells can be seen to circulate in the periphery and inhabit other areas of the skeletal system (leading to lytic lesions) [30,31,32,33]. One study examined SVs, insertions, deletions (indels), or SNVs from the aspirates of focal MM lesions, along with traditional iliac crest biopsies, in 42 newly diagnosed MM patients [34]. Spatial differences in chromosomal aberrations occurred in 40% of cases; however, primary events, such as t(4;14) or t(11;14), were consistently shared between spatial skeletal regions, further confirming that these are indeed primary events. Discrepancies with HRD between spatial regions were also rare. When evaluating SNVs and indels, genomic heterogeneity was more pronounced than that seen with CNAs. The top five genes showing spatial heterogeneity included *NRAS*, *KRAS*, titin (*TTN)*, roundabout guidance receptor 2 (*ROBO2)*, and *BRAF*. 

## 3. Prognosis in MM and Precursor Conditions, Based on Genetic Aberrations

Survival among MM patients is variable and depends on the underlying genetic abnormalities discussed thus far [8]. HRD myeloma tends to have a better prognosis than those cases with IgH translocations [4,17,35,36,37]. Del(17p), del(1p32), t(4;14), t(14;16), t(14;20), and gain 1q are associated with high-risk disease (Table 4) ([38,39]. The inactivation of *TP53,* primarily via del(17p), is associated with a poor prognosis, but the multiallelic inactivation of *TP53* (through *TP53* mutations) is associated with an even worse prognosis [23,24,25]. 

Translocation t(4;14) downregulates *FGFR3* and *MMSET* and, although traditionally thought of as a translocation with a poorer prognosis, this group may be more sensitive to proteosome inhibitors [8,40]. The deletion of the short arm of chromosome 1 (Del(1p)) affects *CDKN2C,* and prognosis may be as detrimental as del(17p) [8,41,42]. Gain (1q) is associated with poor prognosis, perhaps partly due to the increased expression of putative oncogenes, such as *CKS1B* and *MCL*-1 [4,18,42,43]. Monosomy 13 is also associated with poor prognosis, and monoallelic loss of Mir15a/Mir16-1 and may accelerate myeloma progression [44]. *MYC* SVs are associated with progression in MM, and chromothripsis is a strong negative predictor for both PFS and OS [21,22,26,45]. The apolipoprotein B mRNA-editing enzyme catalytic (APOBEC) signature, defined as a unique pattern of variable somatic variants from WES associated with the aberrant activity of APOBEC cytidine deaminases, has been found in all stages of MM, including MGUS and SMM (although to a lesser extent), and is associated with a poor prognosis [46,47].

In the precursor conditions, MGUS and SMM, NGS techniques of serial samples have revealed clonal events occurring at the SMM stage that further evolve as the disease progresses [2]. The characterization of SVs, CNAs, SNVs, and mutational signatures via NGS reveals that clinically stable MGUS and SMM patients harbor a different genetic landscape than those that progress more quickly to MM [48]. These include those genetic myeloma-defining events described above. Those with a lower burden of genetic events had a more benign disease course. *MYC* SVs appear to have a role in the length of time to progression for SMM as well and are typically not found in MGUS patients. For SMM, time to progression may not be significantly higher with non-Ig MYC SVs, whereas those with Ig *MYC* SVs appear to progress rapidly [21]. *DIS3* mutations, in association with Ig *MYC* SVs, may also predict a more rapid progression from SMM to MM. A gain of 1q has been found in approximately 30% of SMM and may be an independent risk factor for progression to MM [18]. 

## 4. Current Molecular Diagnostic Workup for MM, including FISH, NGS, and Mass Spectrometry

Clinical outcomes for MM are dependent on the tumor cell characteristics (measured by cytogenetics and fluorescence via in situ hybridization, FISH) and a laboratory evaluation of the tumor burden (measured by β2 microglobulin, LDH, and albumin) and is referred to as the revised international staging system (R-ISS). Historical methods for diagnosing and monitoring MM disease status include serum protein electrophoresis (SPEP), which detects and quantitates the monoclonal protein (M-protein), and immunofixation electrophoresis (IFE), which detects the M-protein isotype. Serum light chain assays detect the circulating free kappa and lambda light chains [49]. Additional diagnostic workups for MM include a bone-marrow biopsy with immunohistochemistry (IHC) and/or multi-parameter flow cytometry and cytogenetics/FISH to evaluate del(13p), del(17p), t(4;14), t(11;14), t(14;16), t(14;20), 1q gain/amplification, and del(1p).

With emerging therapies and treatment regimens for MM, patients can present deeper and more durable responses than ever before, requiring more sensitive methods for detecting low-level disease or minimal residual disease (MRD). MRD has offered important prognostic information for many hematologic malignancies, and tools to predict MRD have been invaluable. MM patients who achieve MRD have superior survival compared to those who do not [50,51,52,53,54,55,56,57]. The International Myeloma Working Group (IMWG) defines MRD as the persistence or re-emergence of malignant plasma cells, detectable at the level of one malignant cell in at least 1 × 10^5^ normal cells. MRD has been incorporated into the IMWG standardized response criteria [58]. 

Current recommendations for the evaluation of MRD in MM patients are to use either standardized multiparameter flow cytometry (MFC) or NGS assays to detect this level of sensitivity (MRD based on imaging can also be used, but this is outside of the scope of this review) [59]. MFC identifies and quantifies abnormal plasma cells, based on the aberrant protein markers displayed on abnormal myeloma cells. This is achieved by an eight-color flow assay that can analyze 10 million cells. This method for the detection of MRD has been approved by IMWG and can reach a sensitivity of 2 × 10^−6^. The method requires a high level of expertise and samples must be fresh [60]. Adaptive Biotechnology’s clonoSEQ detects clonal immunoglobulin gene rearrangements and is the only NGS MRD method that has been approved by the IMWG. This method requires the identification of immunoglobulin gene rearrangements that are specific for each patient. A disadvantage of NGS for the evaluation of MRD is a long turnaround time when compared to MFC. Its advantage over MFC is that samples can be frozen and stored after genomic DNA extraction for later analysis, and it identifies clonal DNA from cells irrespective of their expression of surface markers, including putative stem cells. Other NGS technologies have recently been developed [61].

Mass spectrometry (MS) is a serum-based method involving the detection of the monoclonal immunoglobulin overproduced by clonal plasma cells. The basis for M-protein detection using MS relies on the antigen-binding region called the complementarity determining region (CDR), which yields an antigen unique to each patient through the process of somatic hypermutation. Each CDR amino acid sequence has a unique peptide sequence and mass that can be detected by MS [61]. Evaluating an intact Ig light chain CDR is the most practical MS approach, specifically, the matrix-assisted laser desorption/ionization time-of-flight (MALDI-TOF) MS. This method has a higher sensitivity for detecting M-protein than SPEP and IFE. In addition, MALDI-TOF has the ability to distinguish monoclonal therapeutic antibodies (e.g., daratumumab) from a patient’s M-protein, which traditional SPEP and IFE cannot do. While this method may be less expensive than NGS or MFC, it can only be performed at facilities with the capability of housing and running the MALDI-TOF MS equipment. 

## 5. The Future of Genomics for MM: Workup and Treatment

Although FISH is primarily used to detect translocations and CNAs, concordance between NGS and FISH may be high, suggesting that, perhaps, NGS technologies can augment the translocations and CNA traditionally detected by FISH and cytogenetics. The consistent use of NGS would allow for additional information on those mutations described above, adding even more value to prognosis and potentially guiding treatment decisions [62]. For example, finding that a patient has both del(17p) and a *TP53* mutation may lead the clinician to be more aggressive with monitoring and/or therapy, knowing that a patient with both these aberrations has a very high-risk disease. In addition, NGS is likely to be more sensitive at detecting MYC rearrangements than FISH [63]. As the cost of NGS techniques continues to decrease, it becomes more feasible to evaluate patients’ RNA through sequencing (RNAseq), which can assess the molecular pathways important for the disease and give additional insights into prognosis [64]. 

In addition to traditional MM therapies, including proteosome inhibitors (Pis), immunomodulatory drugs (IMiDs), and CD-38 monoclonal antibodies, other therapies show improved outcomes for relapsed MM patients, including a SLAMF7 monoclonal antibody (elotuzumab), histone deacylase inhibitors (HDACis including panobinostat and vorinostat), nuclear export inhibitors (Selinexor), antibody-drug conjugates (belantamab), and now chimeric antigen receptor T cell therapies (CAR-T), with the approval of bispecific T cell engager therapy likely to be given soon. However, there are currently no specific therapies targeting mutations in MM. The closest concept of a targetable genetic abnormality and the related drug is venetoclax, a BCL2 inhibitor, which has been shown to benefit about 40–60% of MM patients with t(11;14), likely through BCL2 co-dependence [65,66,67,68]. Gain 1q may also be more resistant to venetoclax, likely through the increased expression of *MCL1* [69,70,71]. As previously mentioned, t(4;14) patients may be more sensitive to PIs, and potential targets of the t(4;14) pathway (*FGFR3* and *MMSET*) have been considered [42,72,73]. Ras pathway inactivation is prevalent in MM, therefore, targeting this pathway has gained some interest; in particular, BRAF inhibitors have been trialed in MM and may show some benefit, but studies have been small [19,73,74]. However, MM tumors are heterogeneous and any successful targeting of the Ras pathway would likely rapidly select for a subclone without dependence on this pathway. Aberrations in *TP53*, *RB1*, and *CDK2NC* are highly pathogenic in MM and predict high-risk disease, but these pathways are difficult to target. Targeting primary events has the potential to yield positive results, as these abnormalities persist through the course of the disease (clonal events), whereas secondary events are considered subclonal; once a specific pathway is targeted, other subclones will persist through other pathways.

Detecting clonal events and potential targets early in the disease process of MM is one of the most appealing future directions. Newer techniques are more sensitive for detecting underlying genetic abnormalities early in the disease process [62]. For example, CNAs may be detectable very early on, at 30 years of age, suggesting the early acquisition of these abnormalities [75]. Although the precursor states, MGUS and SMM, often do not require treatment and may not progress to MM, there are likely to be specific genetic alterations or combinations of such that significantly contribute to progression to a disease state. *MYC* SVs provide an example of how an opportunistic subclone can lead to disease progression and may be a potential target for earlier-stage disease. *MYC* SVs are not present in MGUS but are present in about 25% of SMM and 50% of MM. In addition, MM mouse models have shown that the activation of *MYC* can drive the progression to a monoclonal gammopathy [76]. Further understanding of the underlying genetics and their susceptibility to various medications has the potential to prevent MM, with the ultimate goal of averting the morbidity and mortality of MM. 

## 6. Summary and Conclusions

NGS technologies have revealed the complex nature of MM genetics, further establishing that MM is a disease that is highly dependent on underlying genetic aberrations. Clonal, or primary, events occur early on and are mainly composed of translocations or trisomy of odd chromosomes (HRD), but the acquisition of these primary events remains unknown. Detecting these clonal events at extremely low levels (MRD) is now attainable with the use of NGS and NGF techniques, and we now know that MRD is a prognostic indicator. Secondary, or subclonal, events include those genetic aberrations that occur against a background of clonal events and, overall, are associated with disease progression. Secondary events are more complex and often include the deletion of tumor suppressor genes, complex SVs, and deleterious SNVs. Prognosis and progression are closely tied to specific genetic abnormalities. Although no specific targeted therapy exists for MM, the continued characterization of MM’s clonal and subclonal genetic events, along with the identification of their relative prognostic outcomes, allows for further insights into potential targets. Finally, the ability to halt the disease progression prior to the development of MM is intriguing but will likely not be attainable until we truly understand what makes some cells susceptible to these primary driver events (whether it is random or if there are there underlying changes in the genome that make certain cells more vulnerable to translocations or aneuploidy). Therefore, future research goals should focus on understanding these core principles in MGUS, SMM, and MM.

## Figures and Tables

**Table 1:** Primary/clonal translocation events in multiple myeloma.

Primary Translocations	IgH Translocation Partner	Frequency
t(11;14)	Cyclin D1 (CCND1)	~16%
t(4;14)	FGFR3/MMSET	~15%
t(6;14)	Cyclin D3 (CCND3)	~6%
t(4:16)	MAF	~5%
t(14;20)	MAFB	~2%

**Table 2:** Secondary/sub-clonal copy number abnormalities and secondary translocations in multiple myeloma.

Copy Number Abnormality	Affected Genes	Frequency
Deletion 13q (del(13q))	*RB1, DIS3, MIR15A/MIR16*	~45%
Gain 1q	*MCL1* and *CKS1B*	~40%
Deletion 14q (del(14q))	*TRAF3*	~20%
Deletion 17 p (del(17p))	*TP53*	~8%
Deletion del (1p)	*CDKN2C*	~10%
Secondary Translocations	Translocation Partner	Frequency
Myc	Variable	~25–50%
MAP3K14	Variable	~5%

**Table 3:** Common SNVs and their pathways involved in multiple myeloma.

Pathway	Genes
MEK/ERK signaling	*KRAS*
	*NRAS*
	*BRAF*
	*NF1*
	*PTPN11*
	*FGFR3*
NFkB activation	*TRAF2*
	*TRAF3*
	*CYLD*
	*NFKB2*
	*NFKBIA*
	*BIRC2*
	*BIRC3*
G1/S cell cycle transition	*RB1*
	*CCND1*
	*CDKN2C*
	*CDKN1B*
	*TP53*
RNA processing	*FAM46C*
	*DIS3*
Epigenetic regulators	*DNMT3A*
	*TET2*
	*KDM6A*

**Table 4:** Prognostic primary events in multiple myeloma.

Favorable	High Risk
HRD	del(17p)
	del(1p32)
	t(4;14)
	t(14;16)
	t(14;20)
	gain 1q

## Data Availability

Not applicable.
